# Third wave in India and an update on vaccination: A short communication

**DOI:** 10.1016/j.amsu.2022.103414

**Published:** 2022-03-04

**Authors:** Rusab Baig, Mohammed Abdul Mateen, Abdullahi Tunde Aborode, Shagufta Novman, Isra Abdul Matheen, Ozair Salim Siddiqui, Fatima Abdirazak Ahmed

**Affiliations:** a Shadan Institute of Medical Sciences, Hyderabad, India; b Telangana Institute of Medical Sciences & Research, Telangana, India; c Healthy Africans Platform, Research and Development, Ibadan, Nigeria; d Maharajah Institute of Medical Sciences, Vizianagaram, India; e Ash-Shifa Hospital, Ahmedabad, Gujarat, India; f Kampala University, Garowe, Somalia

**Keywords:** COVID-19, Variant, Transmission, Vaccination, Lockdown, Mutation

## Abstract

The first case of COVID-19 was identified in December 2019. In just two years, the pandemic has wreaked a lot of havoc across the globe. Various determining factors will decide the fate of a looming third wave in India. Although there is no direct evidence of a third wave, many metro cities in India have reported a surge of new cases despite mass vaccination. The rapid development of vaccines and mass vaccination programmes has helped contain the crisis in the past. An updated and robust vaccination campaign along with public measures like the avoidance of large gatherings will help counter the third wave. Over 50% of the eligible population in India has been fully vaccinated to date. The emergence of new strains like the Alpha, Beta, Kappa, and Delta variants of concern, which may exhibit vaccine resistance, may complicate matters. Significant challenges include inadequate data collection, public unawareness, fake news, irregular vaccine supply, and the presence of mutant variants. Comorbidities like dengue complicate disease course. Travel restrictions, personal protective equipment shortages, and barriers to healthcare access are important obstacles to overcome. An increased focus on pandemic preparedness is needed. Targeted vaccination campaigns can help build a favorable public perspective. Data gathering and research need to be promoted. The nation's healthcare policy can benefit from relevant updates based on science and socio-cultural awareness. The fight against the pandemic needs to be cooperative at an international level, with adequate support meted out to resource-poor countries. Long-term structural changes to the healthcare system coupled with strategies for immediate relief will pave the way forward to a stronger system with better contingency planning.

## Abbreviations

COVID-19: Coronavirus Disease 2019
WHO: World Health Organization
GDP: Gross domestic product
H1N1: Hemagglutinin Type 1 Neuraminidase Type 1
NGOs: Non-governmental organization
CCU: Critical care unit
VoC: Variants of Concern
ICMR NIV: Indian Council of Medical Research
NIV: National Institute of Virology (NIV)
IMHE: Institute for Health Metrics and Evaluation
mRNA: Messenger ribonucleic acid
LMWH: Low molecular weight Heparin

## 1 Introduction

COVID-19 (coronavirus disease 2019), an acute respiratory illness caused by the novel coronavirus (SARS-CoV-2, earlier known as 2019-nCoV), has led to an outbreak of pandemic proportions. First identified in Wuhan, China in December 2019, it spread quickly crossing borders becoming a matter of global concern within months. The outbreak was declared by the World Health Organization (WHO) to be a public health emergency of international concern on 30 January 2020, and a pandemic on 11 March 2020 [1].

India's first COVID-19 case was reported on 30th January. There seemed to be no alarming spread observed in February. However, on 4th March, 22 new cases were diagnosed, mainly within an Italian tourist group. Thenceforth, the transmission intensified with new cases identified in large numbers throughout the nation, most of them being linked to people with a history of travel from affected countries. On 12th March, a 76-year-old man who returned from Saudi Arabia became the first fatality. Since then, most of the country has faced the continual spread of infection, albeit at varying rates. The metropolitan cities of New Delhi, Mumbai, Ahmedabad, and Chennai have borne the largest concentration of morbidity [2].

### 1.1 B.1.1.7 or the alpha variant

According to Johns Hopkins data, the Alpha variant first appeared in Southern England in 2020. A study in Lancet reported patients affected by the Alpha variant posed greater CCU admission risk and increased 28-day mortality compared with patients affected by non-alpha strains of the virus [3]. India's first case of the Alpha variant was detected on 29th December 2020. Covaxin, the country's ingenious COVID-19 vaccine was shown to be efficacious against the Alpha variant on 27th January 2021 [4].

### 1.2 B.1.351 or the beta variant

The Beta variant was first detected in South Africa. Findings from Johns Hopkins indicate the potential of the viral strain to re-infect individuals who have recovered from earlier strains of the virus [3]. The first case of beta VoC was detected by ICMR NIV on 5th Feb 2021 and the vaccine efficacy was tested on 7th May 2021 [4].

### 1.3 B.1.617.1 or the kappa variant

According to the World Health Organization, the first samples of the Kappa variant, a double mutant virus, were documented in India in October 2020 [3]. . Genome sequencing of 109 samples at the King George's Medical College in the state of Uttar Pradesh yielded 2 cases of the Kappa variant, with the Delta Plus being detected in most of the remaining samples [4]. Experts estimate roughly 108 crore people, amounting to at least 80% of the country's population, would need to be fully vaccinated to achieve herd immunity. This herd immunity, according to them, is crucial to winning the fight against the pandemic [5]. As of December 2021, over 50% of the eligible population is fully vaccinated.

With the ongoing pace of vaccination in India, is the arrival of the 3rd wave close?

## 2 Body

During the Peloponnesian War around 430 BC, the first pandemic wave was reported [6]. Since then, we've had 16 major pandemics, the most recent being COVID-19. The word “wave” related to disease was first coined in the 15th century, denoting the characteristic “to and fro movement”. The term “pandemic” was first used in 1660, but the phrase “pandemic disease” was first recorded in 1853.

The role of waves in understanding the progress of a pandemic is crucial. There isn't a clear definition of what a pandemic wave is, but it does imply an increase in disease caseload. The WHO (World Health Organization) specified that to announce the end of one wave, the virus has to be brought under control and cases have to fall substantially. As a result, a steady increase in infections is required for a second wave to begin. Furthermore, a standard terminology is essential to aid in the differentiation and understanding of the epidemic and reduces inconsistencies and confusion among healthcare providers, researchers and policymakers [7].

Historically, mankind has seen significant pandemics that lasted for decades or even centuries before they came under control. The Spanish flu pandemic of 1918 is said to have spread in three waves. The third wave was more catastrophic than the first wave but not worse than the second wave. After forty years, a new strain of Asian influenza emerged in 1957. The fatalities were highest among senior citizens and young people. Ten years later, in 1968, a new strain of influenza known as Hong Kong influenza triggered a pandemic in two waves. In April 2009, a new pandemic caused by the H1N1pdm09 (2009 H1N1 Pandemic) virus emerged from Mexico and spread quickly in 3 waves throughout the year, with more severe cases after schools reopened [8].

Throughout history, viral transmission has been through paths used by armed troops and important commercial routes. Globalization has successfully promoted the easy spread of infections that were previously only known to occur in specific geographic areas. As a result, the world is presently in the midst of the pandemic's third wave. In India, there is no direct indication of a third wave, but state-specific waves have been observed. A surge of new cases in Delhi, Kerala, and Maharashtra have been reported, despite mass vaccination. The concept of herd immunity is being complicated by the emergence of new strains and the incidence of vaccine-resistant mutants makes it difficult to predict future disease trends [9]. Mutations produce new variations; nevertheless, data suggests that covid19 mutates slowly due to proofreading enzymes. Although scientists have identified 12,000 mutations, not all of them can spread an infection. If antibody treatments and vaccinations aren't employed properly or if they don't elicit a proper body's natural immune response, dangerous alterations can occur [10].

The course of the third wave is being predicted by wide-base serosurveillance and genomic analysis for any new mutation. In India, 33% of the population is vulnerable. As a result, a large portion of the pandemic can be contained by a major vaccination campaign and the avoidance of large religious and cultural gatherings during the festival season [11]. With the upcoming winter season, seasonal variability in infectious diseases is expected [12]. However, this is less understood, although many hypotheses strongly suggest in its favor [13]. In North Africa, Algeria, Egypt and Libya, including other 20 African countries, have been embracing the third wave since July started. The number of cases reported each day has been more than those recorded during the peak of its second wave [14]. Since the reopening of schools and businesses, there has been a surge in cases. However, one factor contributing to a wave is the emergence of a new viral variant-the latest being the Delta variant. First discovered in India in October 2020, it has now been identified in 92 countries [15]. Many countries, such as Canada, Poland, the Netherlands, Italy, the UK, France, and Germany, are reintroducing lockdowns and travel restrictions given the increase in cases and fear of a third wave [16]. A collapse of Indonesian health care was seen because of the third wave, which saw an average of 5000 new cases each day. Many neighboring countries, such as Pakistan, the Philippines, and Cambodia, are implementing “red zone” lockdowns and increasing the production of oxygen to battle this third wave [17].

Much of what we know about the third wave comes from data gathered by many countries during earlier waves. Our goal should be to enhance preparedness by learning from their pandemic management and avoiding any worsening outcomes. In each round, if the R, or reproduction number, is larger than 1, the number of infected people increases. This is referred to as the epidemic phase. In other words, it tells you how a virus spreads ‘efficiently.’ When a wave is at its peak, it is evident that the virus is spreading at a faster rate. When the wave is receding, however, a tiny increase in R-value can signal the beginning of a new upward trend in the wave [18]. Because of the social and economic losses as well as psychosocial effects caused by the previous two waves, it is critical to confront the impending third wave [19,20].

The virus's spread was contained by imposing a lockdown, which resulted in a historic drop in GDP (Gross domestic product) and employment, with unemployment rising from 8.7 to 23.5% [21]. It can result in a substantial setback to the previously obtained results of national health programmes if not addressed swiftly. If the central government's political goal can be set aside, the pandemic can be contained. Instead of addressing the unprecedented humanitarian disaster, the government-held mass election campaigns overlooked the debilitated health infrastructure. Stringent laws on NGOs (non-governmental organizations) receiving foreign funds have left civil society struggling to respond to the crisis. Strict interpretations of the Foreign Contribution Regulation Act have allowed the government to use it as a “weapon to stifle” civil society by cracking down on organizations it deems unfavorable. Although billions of dollars have been contributed to the PM care funds, the government has been incredibly opaque about how and when they would be used [22].

## 3 Challenges at present

1. No proper information:

The lack of correct data about mortality and morbidity of the viral infection is the biggest challenge concerning the third wave [23]. Underreporting of the number of cases and deaths due to COVID-19 is seen in almost every state of the country and is creating a bias with the epidemiological models, trying to detect the intensity of the upcoming wave. The federal government claims that the death rate is under half a million, whereas models show that it could be 2 to 3 million, or even higher [23].

2. Overconfidence in the general public:

Malls, restaurants, and resorts are full of shoppers and tourists. People are starting to forget the adversity of the second wave, and once again, are in a misconception that the pandemic is over [23].

3. India's drowsy vaccination program:

India tried to increase the vaccine doses by banning its export of vaccines for a month, but that didn't help much [23]. The Central Government asked the vaccine manufacturers to sell their doses at a lower price and allowed them to sell their vaccines to the private healthcare companies at a higher price to compensate. Unfortunately, the private healthcare system didn't get any incentive for the vaccines from the government. Moreover, the government limited the private hospital's profit margins. As a result, only large private healthcare companies are interested in this scheme. The state governments are constantly complaining that they aren't getting vaccines themselves [23].

4. Lack of supply of vaccines:

The mRNA vaccines, Pfizer and Moderna, are more effective against the Delta strain which is a major concern for the third wave in the country [24]. India has been fighting against the two vaccine companies, Pfizer and Moderna, over legal issues [24]. Both the companies want legal protection against any lawsuit which would be filed against them if any serious side effect occurs following vaccination. The companies want this assurance since they developed the vaccines within a few months, and there are higher chances of side effects [24]. The US and UK agreed to the vaccine companies’ terms, but India is reluctant to agree, even if it slows down the pace of vaccination [24]. Moderna and Pfizer have offered around 63.2 million doses, but India wants at least 100 million to even start considering [24].

5. New Variants: ‘The Intelligent Virus’

With more than 130 strains found in the country [25], new ones are forming rapidly.

### 3.1 Delta, Delta Plus and their mutations

The B.1.617.2 also known as the Delta variant, was responsible for the disastrous second wave and is a Variant of Concern (VOC) [26]. The Delta plus variant was earlier classified as a Variant of Concern [27] by the PANGOLIN system of nomenclature [28] and has 2 mutations of the beta and the delta variant and 2 additional mutations, L452R and P871R. According to a study published in the Journal of Immunity, Delta Plus has new mutations in the ORF1a gene, namely, A1146T, P1604L, A3209V, V3718S, and T3750I [29]. The Delta Plus variant is even more concerning than the Delta variant, as it is said to spread 60% faster. Till now, the states of Maharashtra, Kerala, and Madhya Pradesh have been found to have been affected by this variant [27]. Early-stage lung involvement and longer-lasting fever are some of the concerning symptoms of this variant [27].

### 3.2 C.1.2

TIll date, C.1.2 is known to be the most mutated version of the virus. The variant's clinical characteristics and transmissibility haven't been completely understood yet. First detected in South Africa and has been found in China, Congo, Mauritius, New Zealand, Switzerland, Portugal, and the United Kingdom. Many countries like India are still struggling with the new cases emerging due to the highly transmissible Delta variant. N440K and Y449H mutations have been noticed in C.1.2, which tend to escape the antibodies, mainly class 3 neutralizing type. A senior scientist at the National Institute of Biomedical genomics has reported that India needs to be alert for the new variants but there is no need to panic because much information is not available on the variant yet [30].

### 3.3 Epsilon variant

The Epsilon variant identified in California in 2020 has been detected in Pakistan and is a highly transmissible one. This variant seems to be resistant to all the available vaccines [31].

### 3.4 R.1 variant

First identified in Japan, now spreading globally. Till now, more than 10,000 patients in 35 different countries have been affected with the same [32]. Although this new variant is not yet listed by the WHO as a variant of concern or interest, it has a set of mutations that can lead to rapid replication and transmission [32]. Symptoms of the R.1 variant have been found similar to the other strains [32].

6. Dengue and COVID, the deadly combination:

Monsoon, like every year, brings mosquito-borne diseases like dengue and malaria with themselves. Similarly, this year, we have cases of dengue rising rapidly. Northern Uttar Pradesh has become the worst affected state [33]. To make it even worse, both the viral diseases have similar clinical findings, i.e., fever, dry cough, tiredness, sore throat, conjunctivitis, headache, breathlessness, chest pain [34].

Plus, laboratory and biochemical findings have also been found to be similar, which are leukopenia and thrombocytopenia. Moreover, false-positive IgM for Dengue fever has been found in cases of COVID-19 [34]. Despite the similar symptoms, the pharmacotherapy for the 2 is the exact opposite of each other. For example, LMWH is used to prevent the complications of COVID-19, but if used in Dengue cases, it would raise the risk of haemorrhagic disease [35].

## 4 Recommendations

Through evidence-led research and action taken against the COVID-19 pandemic in India, the third wave of the COVID-19 pandemic was absolutely under control through a consistent update on vaccination. However, there is still a need to update and ensure free access to universal health coverage to contain the COVID-19 pandemic crisis effectively and update mass vaccination in India.

Removing practical barriers to immunization is extremely important. Accessibility is one of the greatest barriers that affect immunization intake, especially the vulnerable people due to lack of basic healthcare. In addition to vaccine shortages, personal protective equipment shortages, travel restrictions, the distance to health centers, limited operating hours, and the inconvenient booking system are major challenges. Even simple solutions have the power to make a big difference. For instance, making bookings and travel more convenient can be very impactful.

Think again about vaccination. It is extremely difficult to change skeptical minds regarding vaccines, and misguided approaches such as ‘myth-busting’ may exacerbate the problem of mass vaccination and further spread the COVID-19 pandemic. Instead of creating false information about vaccinations, there is need to spread positive and accurate information about the importance of COVID-19 vaccination.

Vaccination should be presented as a social norm. People tend to do what they perceive as common, so we need to emphasize that vaccination is one of the most widely accepted health activities worldwide. Healthcare workers are an influential and highly trustworthy group of influencers. For instance, Health NGOs in India create a vaccine campaign that encourages people to respect COVID-19 pandemic rules and regulations and bring communities together to share the importance of taking the COVID-19 vaccine. However, more programs and education outreach should increase to avoid another wave of COVID-19 pandemic in India, and also reduce vaccine resistance and hesitancy in India.

Increasing research into different contexts and countries. Almost all evidence comes from high-income settings. In the future, there is a need to improve the evidence base researches in India and other low-and middle-income countries to identify what increases vaccination rates in different countries and why. Vaccination rollout for the COVID-19 vaccine is one of the biggest in history, and it is an opportunity to gather evidence about how to achieve more equitable global access to vaccines, as well as to ensure this work delivers maximum benefits to the people by making global health more equitable by leaving no one behind.

## 5 Conclusion

As the COVID-19 pandemic runs its continual course across the world, the future trajectory of the outbreak in India is difficult to predict. Many elements including the health, humanitarian and socio-economic policies embraced by countries globally will be determining factors in how the pandemic pans out. An international people-centered response grounded in solidarity is vital to recovery.

Primarily, it is important to recognise the absence of a single best strategy. No such singular approach by any country is entirely effective in tackling the COVID-19 crisis. A host of widely varying strategies, some more successful than others, have been employed by different countries. Regardless of the undertaken approach, the pandemic has inflicted significant morbidity and mortality globally on a scale of magnanimous proportions. Ironically, China, initially the epicenter of the pandemic, has been able to demonstrate commendable success in tackling the crisis. However, without independent verification and external scrutiny, any claims of infallible error-free endeavors are rightly met with scientific skepticism.

Additionally, though claims of inaccuracy have been raised on ICMR's statistical modeling, relying upon the Institute for Health Metrics and Evaluation's future mortality estimates for India might not necessarily be the best option either. In the past, IHME's estimates have been notably found to be off-target in the USA. Therefore, ICMR's status as an ingenious national body with a sound field presence warrants a credible standing in policy considerations.

Bloomberg vaccination tracker puts India's vaccination campaign in second place, in terms of doses administered. Despite the challenging prospect of vaccinating such a large population (∼1.3 Billion people), India has put up a valiant effort, achieving one of the highest rates of vaccination across the world.

India faces multifaceted challenges in terms of health provider shortages, poor Internet connectivity, difficult-to-reach terrains, and other socio-cultural factors. Both urban and rural communities in the country will need to work diligently in countering barriers to healthcare access and vaccine intake. Their success in doing so will determine the fate of the dreaded third wave of the COVID-19 pandemic.

As much as technology has helped in disseminating vital information and executing central strategies right down to the grassroots level, it has brought along the vice of misinformation. Widespread propaganda, empowered with social media has created new barriers with no historical precedent to take lessons from. Fake news played a big role in undermining science and faith in public policies. Therefore, novel creative solutions to this problem are crucial to eliminating mistrust in government programmes, especially prevention measures like the vaccination campaign. However, despite all obstacles, there is scope for optimism. Optimal changes in healthcare policy and procedures, engineered with scientific temperament keeping in mind cultural sensitivity can lead to improvements in monitoring, evaluation and healthcare access and usage. This coupled with broader vaccine coverage, can help contain the third wave.

It is important to note that the global effort against the COVID-19 pandemic needs to be cooperative and coordinated. Global support systems need to be put in place in existing international bodies to support countries with insufficient fiscal space in financing social policy and programmes. Elaborate universal social protection systems can be designed, though not without caution to debt sustainability.

It is no secret that the COVID-19 pandemic has not only exposed but also intensified the pre-existent deep-rooted inequalities in the society. In a way, the crisis has provided an unusual opportunity to re-assess current policy architectures and enforce long-term structural changes towards greater social justice. This, coupled with short-term measures to tackle the immediate effects of the crisis, can truly lead to a remarkable human-centered future of administration and public health policy.

## Ethical approval

Research studies involving patients require ethical approval. Please state whether approval has been given, name the relevant ethics committee and the state the reference number for their judgement.

Not applicable.

## Funding

Not funded

## Ethical permission

Not applicable.

## Author's contribution

RB proposed the idea and structured the manuscript. RB, ATA, SN, and MAM did literature reviews. RB, MAM, ATA, SN, ISM, OS wrote the manuscript. OS, MAM, SN and RB worked on the final editing of the manuscript. All the authors made a substantive,intellectual contribution, have read, and approved the final version of the manuscript, and agreed to be accountable for all aspects of the work.

## Consent

Studies on patients or volunteers require ethics committee approval and fully informed written consent which should be documented in the paper.

Authors must obtain written and signed consent to publish a case report from the patient (or, where applicable, the patient's guardian or next of kin) prior to submission. We ask Authors to confirm as part of the submission process that such consent has been obtained, and the manuscript must include a statement to this effect in a consent section at the end of the manuscript, as follows: “Written informed consent was obtained from the patient for publication of this case report and accompanying images. A copy of the written consent is available for review by the Editor-in-Chief of this journal on request”.

Patients have a right to privacy. Patients’ and volunteers' names, initials, or hospital numbers should not be used. Images of patients or volunteers should not be used unless the information is essential for scientific purposes and explicit permission has been given as part of the consent. If such consent is made subject to any conditions, the Editor in Chief must be made aware of all such conditions.

Even where consent has been given, identifying details should be omitted if they are not essential. If identifying characteristics are altered to protect anonymity, such as in genetic pedigrees, authors should provide assurance that alterations do not distort scientific meaning and editors should so note.

## Registration of Research Studies

In accordance with the Declaration of Helsinki 2013, all research involving human participants has to be registered in a publicly accessible database. Please enter the name of the registry and the unique identifying number (UIN) of your study.

You can register any type of research at http://www.researchregistry.com to obtain your UIN if you have not already registered. This is mandatory for human studies only. Trials and certain observational research can also be registered elsewhere such as: ClinicalTrials.gov or ISRCTN or numerous other registries.

1. Name of the registry:
2. Unique Identifying number or registration ID:
3. Hyperlink to your specific registration (must be publicly accessible and will be checked):

## Guarantor

The Guarantor is the one or more people who accept full responsibility for the work and/or the conduct of the study, had access to the data, and controlled the decision to publish.

## Declaration of competing interest

The authors declare no conflicts of interest.
